# Community Perceptions of Community Health Workers (CHWs) and Their Roles in Management for HIV, Tuberculosis and Hypertension in Western Kenya

**DOI:** 10.1371/journal.pone.0149412

**Published:** 2016-02-22

**Authors:** Beth Rachlis, Violet Naanyu, Juddy Wachira, Becky Genberg, Beatrice Koech, Regina Kamene, Jackie Akinyi, Paula Braitstein

**Affiliations:** 1 Academic Model Providing Access to Healthcare (AMPATH), Eldoret, Kenya; 2 University of Toronto, Dalla Lana School of Public Health, Toronto, Ontario, Canada; 3 The Ontario HIV Treatment Network, Toronto, Ontario, Canada; 4 Department of Behavioral Sciences, School of Medicine, College of Health Sciences, Moi University, Eldoret, Kenya; 5 Department of Health Services, Policy and Practice, Brown University, Providence, Rhode Island, United States; 6 Department of Medicine, School of Medicine, College of Health Sciences, Moi University, Eldoret, Kenya; Harvard Medical School, UNITED STATES

## Abstract

Given shortages of health care providers and a rise in the number of people living with both communicable and non-communicable diseases, Community Health Workers (CHWs) are increasingly incorporated into health care programs. We sought to explore community perceptions of CHWs including perceptions of their roles in chronic disease management as part of the Academic Model Providing Access to Healthcare Program (AMPATH) in western Kenya. In depth interviews and focus group discussions were conducted between July 2012 and August 2013. Study participants were purposively sampled from three AMPATH sites: Chulaimbo, Teso and Turbo, and included patients within the AMPATH program receiving HIV, tuberculosis (TB), and hypertension (HTN) care, as well as caregivers of children with HIV, community leaders, and health care workers. Participants were asked to describe their perceptions of AMPATH CHWs, including identifying the various roles they play in engagement in care for chronic diseases including HIV, TB and HTN. Data was coded and various themes were identified. We organized the concepts and themes generated using the Andersen-Newman Framework of Health Services Utilization and considering CHWs as a potential enabling resource. A total of 207 participants including 110 individuals living with HIV (n = 50), TB (n = 39), or HTN (n = 21); 24 caregivers; 10 community leaders; and 34 healthcare providers participated. Participants identified several roles for CHWs including promoting primary care, encouraging testing, providing education and facilitating engagement in care. While various facilitating aspects of CHWs were uncovered, several barriers of CHW care were raised, including issues with training and confidentiality. Suggested resources to help CHWs improve their services were also described. Our findings suggest that CHWs can act as catalysts and role models by empowering members of their communities with increased knowledge and support.

## Introduction

High healthcare costs, a lack of adequate infrastructure, and health worker shortages all decrease the ability of Low- and Middle-Income Countries (LMICs) to deliver primary healthcare services to their populations [1–3]. Sub- Saharan Africa has just 3% of the global health workforce [4] and an estimated 1.5 million more health workers are needed just to be able to provide basic health services in the region [5]. Largely in response to these health worker shortages, the World Health Organization (WHO) launched the “treat, train, retain” initiative in 2006 [6] in an effort to strengthen and expand the global health workforce. This included the development of more formal cadres of Community Health Workers (CHWs), defined as “members of, selected by, and answerable to the communities where they work; supported by the health system; and receiving less training than formally trained health workers” [7, 8]. In general, CHWs deliver low cost primary healthcare services to the communities they serve [9]. They are well situated to bridge the gap between communities and the healthcare system, and can facilitate engagement in care and overall continuity of care [9, 10]. CHW programs range from large-scale national programmes to smaller community-led initiatives [1, 11] and have led to improved access and coverage of health services in rural and remote areas in LMICs [11, 12]. Indeed the 2008 WHO report *Primary Health care- Now More Than Ever*, citing previous “failures and shortcomings” that have resulted in massive health inequities between and within countries, reinforced the important role of primary healthcare and CHWs in ensuring health for all [13]

While communicable diseases like HIV/AIDS and Tuberculosis (TB) continue to exert a heavy toll in LMICs [14, 15], non-communicable diseases (NCDs) are also on the rise. An estimated 639 million (625–654 million) hypertensive individuals now live in LMICs [16, 17]. In Kenya (population: 44.35 million [18]), NCDs increasingly account for a higher proportion of national morbidity and mortality [19, 20] and numerous people are now living with more than one chronic condition. For example, while people living with HIV (PLWH) represent approximately 7.1% of the adult Kenyan population, HIV/TB co-infection is estimated to affect 48% of all new TB patients [21] and hypertension among PLWH is estimated at 11.2% and 7.4% for men and women respectively [22].

As a result of the rise in comorbidities, many settings, including Kenya, have begun to provide integrated chronic care [23–25]. Although there are differences in the etiology, treatment and prognosis across various diseases, in the case of HIV, TB and hypertension (HTN), all require regular engagement in the healthcare system for proper treatment and management. Linkage to formal care following a diagnosis is a critical for maximizing health outcomes, reducing morbidity and mortality, and in the case of communicable diseases like HIV and TB, minimizing the risk of forward transmission [26–31].

In part given that the number of physicians (0.18) and nurses/midwifes (0.79) per 1,000 Kenya is well below the recommended critical workforce [32], a national CHW program was launched in 2006 as part of a broader community health strategy. The goal of the CHW program is to ensure every household has access to primary care [11, 33]. Through proper training, CHWs can test for various illnesses and follow-up with individuals in their communities to ensure their conditions are being properly managed. This may occur at the household-level through routine follow-up or by ensuring individuals in communities are properly linked to the health facility where treatment and management is available [34]. Although CHWs are well situated to encourage timely engagement in care and thus can play an important role in the management of various chronic conditions, it is unclear how they are perceived by the communities they serve. Negative perceptions of CHWs may impact their effectiveness at supporting linkage and engagement with chronic disease care and management. We, therefore, conducted a qualitative study to explore perceptions of patients, caregivers, community leaders and healthcare workers, including perceptions of CHWs role in chronic disease management. While we focused primarily on HIV, TB and HTN care in western Kenya, here engagement in care is discussed more broadly.

## Materials and Methods

### Study setting

The AMPATH Consortium, based in Eldoret, Kenya (about 350km north-west of Nairobi) was initiated in 2001 as a joint partnership between Moi University School of Medicine, Moi Teaching and Referral Hospital (MTRH) [35, 36], and a consortium of North American universities led by Indiana University School of Medicine. With financial support from United States Agency for International Development (USAID), the USAID-AMPATH Partnership was established in 2004. The history, organizational structure, and health programs of AMPATH have been described elsewhere [35, 37]. The AMPATH Consortium provides technical support, mentorship and training to Kenyan medical faculty and staff with the aim of developing healthcare services in Kenya. AMPATH delivers care, provides education, and performs research in networks of urban and rural Ministry of Health hospitals, health centers, and dispensaries in western Kenya. The initial goal of the program was to establish an HIV care system to serve the needs of both urban and rural patients as well as to assess the outcomes and barriers of antiretroviral therapy (ART). AMPATH has enrolled over 160,000 HIV-infected adults and children plus 21,000 HIV-exposed infants in >65 Ministry of Health facilities around western Kenya. Currently, >85,000 patients are actively followed, 83% of whom are on combination ART; 21% are aged ≤14 years. All HIV and tuberculosis (TB)-related care and treatment are free at the point of service for patients. Patients are managed according to National Kenyan protocols, which are consistent with WHO guidelines. While AMPATH initially focused on patients infected with HIV, it has since expanded to provide primary health care, maternal and child health services and chronic disease management (specifically diabetes and hypertension) to a catchment population of over 2 million persons [26]. This study was undertaken in three AMPATH sites, namely Chulaimbo, Teso and Turbo.

### Current Role of CHWs within Kenya and AMPATH

CHWs are volunteers who are recruited from the community with input from Community Health Extension Workers (CHEWs) and members from the village, sub-location and district. They are often recruited through a baraza (i.e., a meeting with community elders) [34]. Ideally, CHWs should be literate and respected so that they can help to motivate others in their communities. In general, they receive approximately 6 weeks of initial training and quarterly refresher training [34]. In the current Kenyan government model, CHWs are supervised by a facility-based and government-employed CHEW. Each community unit, made up of approximately 5000 people is supported by 50 CHWs and 2 CHEWs. Each CHEW supervises approximately 25 CHWs [34]. CHEWs support CHW through supervision and coaching and meet with their CHWs ideally monthly [38].

CHWS perform numerous tasks both in households but also in the community at large. Essentially the role of CHW is to identify health needs, educate and manage some conditions at the household level and link/refer patients to health facilities. Their main tasks include disease prevention and control, family health services and hygiene and sanitation [39]. Specific tasks include taking vital signs, dispensing meds, providing individual and group education, community mobilization, and advising on proper diet/nutrition and sanitation/hygiene. Other tasks may include defaulter tracing, raising awareness of NCD control, caring for the chronically ill, and health promotion. CHWs are also trained on aspects related to community and household entry and data collection methods. Currently within AMPATH CHWs are considered volunteers, although at the time of the study were receiving 2000 Kenyan Shillings per month (~$20 USD) for overseeing 50 households. CHWs also received training certificates. At the time of this study, AMPATH worked with CHWs who were trained to do home-based HIV testing and some able to take blood pressure. Point of care testing has always been available at health facilities, however it has primarily been performed by facility-based providers (e.g., nurses and clinical officers) rather than CHWs.

### Study Population

This study targeted patients within the AMPATH program including patients receiving HIV, TB, and HTN care, as well as caregivers of children with HIV, community leaders (religious leaders, traditional healers, village elders, assistant chiefs), and healthcare workers including the AMPATH safety net team (Nutritionist, Psychosocial, Outreach, Social Work teams) and providers (AMPATH clinical team, Ministry of Health staff).

### Study design

This was an exploratory qualitative study conducted between July 2012 and August 2013. The goal of the study was to understand the role of CHWs in linkage and retention, what they need to do their work and how communities perceive them. Study participants were purposively sampled from the three AMPATH sites: Chulaimbo, Teso and Turbo. Specifically, individuals were recruited if they could provide different perspectives on CHWs and their roles, could share their experiences, behaviours and perceptions, and have an understanding of the cultural and societal context. Individuals needed to be a resident of one of the three catchment areas. In-depth interviews and focus group discussions (FGDs) were used to collect data. We conducted a total of 16 in-depth interviews and 26 FGDs. Tables 1 and 2 shows the distribution of participants per site. FGD were utilized only for patient groups as they were considered a more homogenous group. In-depth interviews were held with community leaders and provider groups only as they were considered a more heterogeneous group that was purposely selected based on their unique and comprehensive knowledge on the topics relevant for the present study.

**Table 1:** Distribution of focus group discussion by site (n = 26).

Site	PLWH		HTN		TB		Caregiver	Safety Nets	HCW
	Men	Women	Men	Women	Men	Women	All women	Mixed	Mixed
**Chulaimbo**	1	1	-		-		1	1	1
**Teso**	1	1	1 (mixed)		1 (mixed)		1	1	1
**Turbo**	1	1	1		1	1	1	1	1

PLWH = People Living with HIV; HTN = hypertensive patients; TB = TB Patients; Caregiver = for children living with HIV; Safety Nets = includes nutritionists, outreach workers, social workers, psychosocial works; HCW = Health Care Worker including clinical officers, nurses, pharmacists and lab technicians.

**Table 2:** Distribution of in-depth interviews by site (n = 16).

Site	Religious leaders	Traditional healers	AMPATH/Primary Care worker (in charge)	Ministry of Health worker (in charge)	Village elder/assistant chief
**Chulaimbo**	1	2	1	1	1
**Teso**	1	1	1	1	1
**Turbo**	1	1	1	1	1

Participants were asked to describe their perceptions of *AMPATH CHWs* including identifying the various roles they play in terms of chronic disease management including engagement in care for HIV, TB and HTN. A question guide was developed and individuals were asked: What is your perception of the AMPATH CHWs as linkage-to-care facilitators (Probes: confidentiality, what information do they give? Is there enough education?). As well, participants were asked: What linkage information do you think should be given by the CHWS at the community-level (Probes: What kinds of resources should they use? What information should resources contain?). Related to this was exploring positive and negative attributes of CHWs as well as identifying the resources CHWs need to be able to effectively link individuals to care. In addition, basic socio-demographic information including age, gender, educational level and occupation was collected. Trained research assistants identified the target groups at AMPATH health facilities and informed them about the study. Health facility in-charges assisted with contacting the participants. The interview sessions and FGDs took approximately 1 hour and were conducted in either, English, Swahili, Kalenjin, or Luo. All sessions were audio recorded and for the FGDs, scribes also recorded session proceedings. At the end of each session participants were provided with transport reimbursement of 200 Kenyan Shillings.

This research was program driven and was situated within the broader AMPATH Care Program with the goal of improving linkage and retention of patients within existing clinics. It was considered a low-risk rapid appraisal. Consent was obtained prior to beginning data collection and again prior to commencing audio recording. While consent forms were not used, transcripts from the FGDs and in-depth interviews demonstrate agreement and consent to proceed with the data collection. Note that ethical approval for this study was obtained through an amendment of a larger AMPATH Program protocol that received ethical approval from the Institutional Research and Ethics Committee (IREC) of Moi University College of Health Sciences and Moi Teaching and Referral Hospital as well as the Indiana University Institutional Review Board (IRB).

### Data Analyses

**Fig 1:**
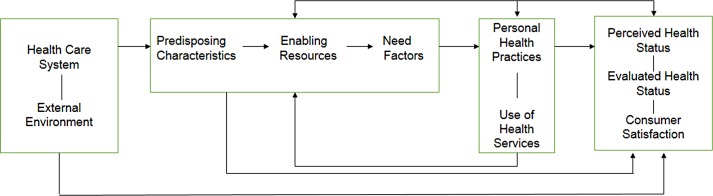
Andersen-Newman Framework for Health Services Utilization.

Recorded interviews were transcribed and translated to English. The data were then coded and themes related to general perceptions of CHWs, perceptions of CHW roles and resources used by CHWs to facilitate engagement in care were identified. Ideas from different interviews were pooled together and integrated into common themes. Concepts from these themes were generated and we used a conceptual model based on the Andersen-Newman Framework of Health Services Utilization to organize the presentation of the results. In the Andersen Newman Framework (Fig 1), an individual’s access to and use of healthcare is a function of three main factors: 1) Predisposing Characteristics (socio-cultural characteristics of individuals that exist prior to their illness); 2) Enabling Resources (the logistical aspects of obtaining care, which can include personal, family and community resources); and 3) Need Factors (the most immediate cause of healthcare use from problems that generate the need for care) [40]. In this analysis, CHWs are viewed as a potential enabling resource and we organized the findings based on positive and negative perceptions of CHWs as either enabling (i.e., facilitators) or inhibiting effective management of HIV, TB and HTN (i.e., barriers), respectively. For validation, independent coding and identification of themes were conducted by five investigators. We started with a codebook that had *a priori* codes that were derived from the original question guide. The 5 investigators worked independently to identify emerging inductive codes that were then added to the codebook as necessary although data was also interpreted based on pre-existing knowledge about the context, the study objectives and the identified themes. Training relating to qualitative data analysis including coding and thematic analysis was also provided. All had extensive experience collecting and analyzing qualitative data. Note that no software was used. The final write up consisted of summaries, interpretations and textual excerpts.

## Results

### Characteristics of Participants

A total of 207 participants including 110 individuals living with HIV (n = 50), TB (n = 39), or hypertension (n = 21); 24 caregivers; 10 community leaders; and 34 healthcare workers participated in the study.

### General community perceptions of CHWs in chronic disease management

Perceptions regarding CHWs level of involvement with the health care system varied within our sample. In our setting, many perceived CHWs to be just like peers (i.e., as patients). Participants identified several key roles of CHWs in communities including promoting primary health care services, encouraging testing for various conditions, and demystifying hospital care: “*The CHWs can easily identify symptoms of various diseases like malaria*, *high blood pressure but an herbalist doesn’t”* -Traditional healer, Chulaimbo. *“They tell the community to come for HIV testing and generally about hygiene*. *All kinds of prevention methods; for example cleaning the compound to avoid mosquitoes*”–Provider, Chulaimbo

In addition to providing adequate information about HIV and TB, CHWs also encourage awareness about HIV and TB and prevention and treatment. “*They educate people on how TB is spread*- Traditional healer, Chulaimbo. *“They start with education before testing*…*They give enough education on prevention and treatment*”–Religious Leader, Chulaimbo

CHWs may also be integral to linkage to care: “*CHWs have contributed a lot to service provision*, *facility linkage and also de-stigmatization”*-Traditional healer, Chulaimbo. *“She talked to me very well until I decided to come here because I know that it is my life and that of my child*.*”* -Caregiver, Chulaimbo. Related to this is retention of patients in care including acting as adherence supporters and reminders for healthcare seeking behaviours as one participant noted: *“In case one forgets*, *they act as the reminder*” -PLWH, Chulaimbo

CHWs also make regular home visits and reach out to remote villages: “*They bring information to the household*…*when they walk around the village and teach*, *people gather courage and come for medication*”- TB patient, Chulaimbo

Finally, several participants in our study had never been in contact with a CHW and suggested that they are unknown in some areas: *“I have never seen them”* -TB patient, Chulaimbo

### CHWs as an Enabling Resource to Management of HIV, HTN and TB

#### Facilitators

Participants identified various positive aspects of CHWs that facilitate their role in effective management of chronic diseases. Participants felt that CHWs generally do a good job. One caregiver in Teso noted that “*They are good and do good service*…*”* A traditional healer in Turbo stated that *“*…*they approach people in a good way*. *They counsel people*, *especially those that have not come out until they accept to be tested*. *They are people (CHWs) that have sacrificed and they have skills”*-Caregiver, Teso “*Mostly the CHWs are good*. *They visit us at home and when they find a patient who has not gone for medical attention*, *they advise that person to be taken to the hospital*…*Since they have undergone training*, *they can easily identify signs of HIV*…*One is then diagnosed with either HIV or TB in the hospital”* -Traditional healer, Chulaimbo.

Some participants felt that the CHWs are good at maintaining confidentiality. One participant from Teso stated that the CHW supervisors–Community Health Extension Workers–have never reported any ethical issues regarding confidentiality: “. …*I get information from the Community Health Extension Workers but so far have not heard of a breach in confidentiality*”- Provider, Teso. “*Yes*, *they are confidential*. *I have never heard anything from the ones who visit me*”- TB patient, Chulaimbo. This was supported by another individual who noted: “*They don’t carry information from one household to another”* -TB patient, Chulaimbo

In general, participants found value in CHWs. A participant in Chulaimbo said: “*I can talk about them because they usually visit me*. *Given my health condition*. …*they usually come and check my blood pressure and find out how I am doing*”- TB patient, Chulaimbo. A religious leader described how their work and communication style is viewed by local communities: “*the community appreciates their work*. *They have a good approach*”-Religious leader, Chulaimbo.

#### Barriers

Some participants felt that CHWs are a nuisance to community leaders: “*There is a youth at our place who is a health worker and a drunkard*. *When he comes he says*, *‘You see I have come to monitor your health*, *buy me a glass (several laughs)*. *He wants you to know that he is helping you yet he is on duty and is being paid*.” -Caregiver, Turbo. Another participant stated: “. …*they are selective of the homes they visit*…*they go where they can be given food; they can’t go to a home that does not offer morning tea*. *When they spot a banana in your homestead they will ask for it*…*once he tells you to use a condom*, *he starts eyeing the avocado tree (several laugh)*” -TB patient (in FGD), Chulaimbo

While some participants felt that CHWs do a good job with maintaining confidentiality, other participants felt that CHWs can sometimes compromise confidentiality which may inhibit their ability to effectively enable linkage to care and management of chronic diseases. This was expressed by community leaders and healthcare providers alike: “*Some people may not be confidential because as I said*, *they are not equipped with knowledge on how to keep information and how to pass it*” Provider, Chulaimbo. “*They keep secret but not 100% because sometimes a person may be tested and within some period people know that that person was tested and found with HIV*. *How did this information reach the public*? *So confidentiality was not adhered to*” Community leader, Turbo. One participant argued some people may be better than others at maintaining confidentiality: “*These people are in two categories*. *The young do not keep secrets*. *When we are in meetings they have those machines [phones] that they ring and ask*, *‘How are the results of this person*?*’*…*They don’t keep secrets*” -HTN Patient, Teso

CHWs can also cause fear and as a result may discourage individuals from being tested for various illnesses in the first place. A TB patient from Chulaimbo stated “*I understand some people may hide from them*.” This may relate to a fear of being recognized by others in their communities as being “sick” or “infected”. One PLWA in Chulaimbo stated that stigma can be felt particularly “*when a CHW is from your village*”.

Some participants felt that CHWs don’t always have adequate health information on all the relevant diseases and health issues which may be considered a barrier to linkage and management of care: “*Most of them (CHWs) I know only deal with HIV/AIDS*. *I don’t think they have enough education*, *so I was afraid to be tested by them*. *I know them*, *they don’t have enough education*. *I wondered how they’d help me*. *I questioned the syringes they had*, *if they are contaminated or not (all laugh)”* HTN group, Turbo. A religious leader noted: *“…*.*there is that notion that CHWs from AMPATH deal with HIV so HTN and TB come in later*” Religious leader, Teso.

Some participants suggest that the lack of information among CHWs may stem from little education and/or a lack of formal training: “*Some of the workers are clients themselves and they are just school leavers*. *No professional training*. *They may not address issues to do with health adequately*”- Provider, Chulaimbo. Participants also described situations where CHWs do not always do their jobs or assist when their help is needed: “*I usually see them but when one is seriously ill*, *they don’t assist so I don’t understand their role*. …”–PLWH, Teso.

Alternatively, some participants indicated that CHWs can raise patient expectations regarding donor support/funding/gifts that may be available for them. Instead of developing positive and sustainable ways to manage their own health, individuals may become too reliant on monetary incentives. A TB patient in Chulaimbo noted that “*they are doing a good job because there are places where they give people money after testing and this motivates them*. …” -TB patient, Chulaimbo.

#### Suggestions for improving CHWs as an enabling resource

Participants were asked what information should be given to CHWs in order for them to be able to positively improve engagement. Several resources and tools were suggested. In addition to posters and charts describing key and useful information, participants also suggested that pamphlets, brochures and fliers could be produced and handed out to community members. A traditional healer in Teso noted the need for “*any information that could be put on paper that communicates about these conditions*.” However, as another participant pointed out, other mediums of communication may be needed: “*brochures*, *pamphlets should be minor and not big*…*because the reading culture…is not all that good*.”- Religious leader, Teso. Participants felt that the role of CHWs could be expanded beyond the household with more interactive events and sessions held within the larger community. Suggested avenues for CHWs to work and disseminate information were also provided and included: *mabaraza* (community gatherings), schools, markets/shopping malls, churches, youth groups, and *chamas* (associations/party/guilds). One participant noted that: “…*these barazasas are the best place to give information*” (Provider, Turbo). Participants also suggested that CHWs could also educate communities through theatre and radio (including tape recorded messages).

## Discussion and Conclusions

In the present study, we explored the role of CHWs generally, and in terms of facilitating engagement in care for chronic disease management in western Kenya. In this analysis, we viewed CHWs as potential enablers to care engagement for chronic diseases. General perceptions of CHWs were identified including factors that may facilitate or inhibit their ability of CHWs to link and engage the communities they serve with proper disease management.

We believe that CHWs can act as catalysts and role models by empowering members of their communities with increased knowledge and support. Indeed, the findings of the present study suggest that, generally, CHWs are well received in the communities they serve and have the capacity to promote awareness and positive health-seeking behaviours. CHWs are viewed by the communities as an important link in the health system [41]. Participants in the present study discussed various roles for grass root CHWs including the promotion of primary health care and the generation of awareness about the relevant health issues affecting the communities in which they serve. They are in an ideal position to bridge the gap between individuals and health facilities [42, 43]. Given their proximity to the communities in which they work and in the case of Kenya, they live, CHWs are well placed to break down social barriers and make health information interpretable and comprehensible to their peers [43–45]. In this way, they are able to “demystify” the healthcare system, and as a result, successfully encourage linkage and uptake of services [25, 42, 46, 47] for those who may not have otherwise engaged. Importantly, a lack of knowledge and/or preconceived fears regarding the health care system may affect positive health-seeking behaviours. Indeed, previous research suggests that CHWs and other community-based interventions can make testing for HIV and TB more accessible to historically underserved populations [47, 48].

Importantly, participant perceptions of CHWs indicates that some attributes of CHWs may actually hinder effective management of chronic diseases. For example, numerous barriers of CHWs were identified including issues related to poor confidentiality and a lack of information on relevant health issues. Some participants expressed concerns around whether CHWs are able to keep information confidential. The issue of confidentiality is particularly important when CHWs live in the communities they serve. Furthermore, given concerns of stigma, either real or perceived, and fears of non-intentional disclosure particularly in the case of HIV, maintaining patient confidentiality is not only ethical but critical [49] and can encourage positive experiences with the health care system. Related to this is the ways in which CHWs are communicating with their clients. As some participants pointed out, CHWs can, in certain situations, be a “nuisance” to communities and are perceived as taking advantage of clients during home visits. This was particularly noted among community leaders. One study in our setting noted that while CHWs are generally well accepted, they may face challenges when conducting regular home visits particularly when the communities they are engaging with perceive that they have no useful resources available to provide [39]. In this fashion, they may also be perceived as a nuisance although this may also stem from who CHWs are and how they are trained.

Enhanced training on common diseases and health care ethics is needed for CHWs in our setting. While some participants spoke positively about the level of awareness CHWs are able to generate around various diseases, others noted that the CHWs may not always have accurate or even reliable information. Unfortunately, this can lead to misconceptions about prevention strategies, the severity of an illness, and the need for engagement in the healthcare system. As a result, individuals may either believe they do not need and/or choose not to engage to care, resulting in poor health and clinical outcomes. Indeed, participants in our study suggested that training CHWs to administer diagnostic tests results in timelier engagement in appropriate care [50]. Education for CHWs include training on the more logistical aspects of their jobs including but not limited to household entry, community sensitization, data collection and recording, and relevant ethical issues (i.e., how to maintain confidentiality). Shifting to information gathering tasks could also improve timeliness of data [11] and can also enhance the way CHWs are viewed in their communities [41]. It is worth noting that issues related to inadequate training have been reported in other studies in our setting [51, 52]. Interestingly, the ability to be recognized by their peers can also help to motivate CHWs and have a positive influence on their own attitudes towards their jobs [11, 52].

As noted above, individuals who are recruited to be CHWs should be well respected members of a community whom are able to motivate others. But the information and training they receive is a critical component of how they are able to effectively complete their tasks. As CHW programs become integrated more formally into exiting healthcare systems, there is a need for comprehensive education and training to ensure that the CHWs are disseminating appropriate and accurate information into their communities. Related to this is how and where information is disseminated. Numerous strategies and venues were suggested for CHWs as a way to improve their ability to disseminate information to the communities they work in and essentially link them into care. Simple messages in the form of posters, pamphlets, brochures and fliers could be produced and handed out to disseminate accurate and relevant information about key health conditions affecting their communities. Participants noted that venues where communities gather such as mabarazas and churches should be a target spot for CHWs to work in.

There are a few limitations worth noting. This was a qualitative study and we acknowledge that our findings cannot be generalized to the wider Kenyan population. It mainly presented views of communities studied and was limited to perceptions about AMPATH health care. The views presented in the present study may not represent the views of other communities not included in the present study. There may be other perceptions and attitudes that were not captured but that are relevant for engagement in care. As CHWs are embedded within their communities, some participants may not have responded honestly given interpersonal relationships they may have with the CHWs working there.

In conclusion, we believe that CHWs can act as catalysts and role models by empowering members of their communities with increased knowledge and support. While various facilitating aspects of CHWs were uncovered, several barriers of CHW care were raised. In addition, participants described resources that are needed for CHWs to better act as an enabling resource for linkage and engagement in chronic disease management. Our findings have important implications for other CHWs programs working with communities to promoting positive health seeking behaviours including successful linkage and retention in care.
